# Salvaging the Digit in Invasive Subungual Malignancies Using a Triple Technique under Awake Local Anesthesia

**DOI:** 10.1155/2021/4648627

**Published:** 2021-09-30

**Authors:** Samer Abdel Al, Mohamad K. Abou Chaar, Ala'a Aldeen Alkhatib, Muhamad Al-Qawasmi, Mohammad Barham, Sameer Yaser, Samer Salah, Abed Al Raheem Suleiman, Wafa Asha

**Affiliations:** ^1^Department of Orthopedic, Hand and Microsurgery, King Hussein Cancer Center, Amman, Jordan; ^2^Department of Surgery, King Hussein Cancer Center, Amman, Jordan; ^3^Department of Medical Oncology, King Hussein Cancer Center, Amman, Jordan; ^4^Department of Research, King Hussein Cancer Center, Jordan; ^5^Department of Radiation Oncology, King Hussein Cancer Center, Jordan

## Abstract

**Introduction:**

Amputation for subungual malignancy (SUM) was thought to be the gold standard in preventing recurrence and metastasis. The rationale behind this aggressive treatment was never based on scientific evidence. Even though multiple recent studies supported more conservative management by illustrating successful results of the digit salvage technique, especially for “in situ” SUM, this salvage approach is not well supported for the more aggressive type of the “invasive” SUM; herein, we salvaged two cases of “invasive” SUM. *Case Presentation*. We present two cases of invasive SUM without radiographic evidence of intraosseous involvement, where we avoided digit amputation for both invasive subungual squamous cell carcinoma of the thumb and invasive subungual melanoma of the ring finger. Both were salvaged by using a triple technique under awake local anesthesia which included (I) radical excision of the nail bed unit including both eponychium and periosteum, (II) dorsal cortical bone shaving using a high-speed burr for the distal phalanx, and (III) flap coverage. Brunelli flap was used for the thumb in the first case, and V-Y plasty combined with proximal nail fold advancement flap was used for the ring finger in the second case. There was no evidence of local or distant recurrence, with a good functional outcome after 2.5 years in the first case and 2 years in the second.

**Conclusion:**

Ensuring complete resection with negative margins while preserving the functionality of the affected digit is considered to be the optimal challenge in treating “invasive” subungual malignancies. These two case reports contribute by reporting a successful digit salvage. The safety of this procedure could be confirmed by larger series and longer follow-up periods.

## 1. Introduction

“In situ” subungual malignancy (SUM) has been treated with distal phalanx amputation of the digit because aggressive surgery is only appropriate for what was considered to be an aggressive malignancy. After questioning the efficacy of radically amputating the digit involved with in situ SUM, a debate about proper surgical management recently erupted. There is no consensus on the recommended level of excision or type of coverage as of yet; however, once the bone invasion is detected, radical amputation is the mainstay of treatment, reaching the level of the first unaffected joint [1]. Because of the morphology and functional requirements of the nail unit, SUM in this location necessitates a sophisticated surgical technique for management and reconstruction. Reconstructive options may differ depending on the size and location of the lesion [2]. The challenges of salvaging “invasive” SUM are greater than those of salvaging “in situ” SUM, which requires more radical excision, as shown in our two cases.

## 2. Case Presentation

### 2.1. Patient 1

The first patient is a 54-year-old right-handed male smoker (1 pack per day for 25 years), with a body mass index (BMI) of 20.35 kg/m^2^. He has negative medical and surgical histories but has a positive family history of lung cancer in his brother. Almost three years ago, he started to complain of discoloration in the distal right thumb associated with nail pain. He sought medical attention outside our facility and underwent multiple nail bed partial debridement procedures as it was thought to be an infection due to a foreign body. After the third procedure, a shaving biopsy was taken to rule out malignancy, and it came back as invasive squamous cell carcinoma (SCC) with an involved deep margin. The patient was referred to King Hussein Cancer Center (KHCC), for further management. On presentation, his hemoglobin (Hb) was 14.8 g/dl, his white blood cell (WBC) count was 7.3·10^3^/*μ*l, platelets were 262·10^3^/*μ*l, creatinine was 0.71 mg/dl, and albumin was 4.38 g/dl. On examination, a healed nail bed was noticed, residual changes were detected with a discolored and deformed nail bed due to multiple bone procedures. Magnetic resonance imaging (MRI) with intravenous contrast showed ill-defined soft tissue thickening with linear enhancement over the nail bed, without any definable nodules, masses, or bone invasion; also, no palpable lymph nodes were found. Positron emission tomography/computed tomography (PET/CT) scan revealed a small focus of mild uptake (SUVmax = 1) in the right distal thumb which mostly represents postinterventional reactive changes, but still, a residual tumor could not be ruled out; other than this, no lymphadenopathy and no metastasis were found. The patient and his family were properly counseled and informed about the multidisciplinary clinic (MDC) decision, which included a radical excision of the nail bed including both eponychium and periosteum and bone shaving of the dorsal bone of the distal phalanx because the nail bed had been deformed by multiple procedures, and deep margins were involved upon pathology review of the previously excised specimen, followed by a soft tissue reconstruction to cover the exposed bone by the Brunelli flap.

A handheld ultrasound probe was used to check the dorsoulnar artery perioperatively.

**Figure 1:**
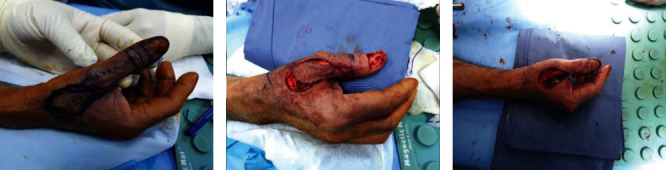
Intraoperative images showing the first stage of the Brunelli flap. Intraoperative image showing (a) marking of the Brunelli flap and the dorsoulnar artery perioperatively, as well as the palmar connection between the dorsoulnar artery and the palmar ulnar digital artery at the level of the interphalangeal joint of the thumb. (b) The skin paddle is incised deep till the paratenon of the extensor pollicis longus on its radial edge is reached and deep to the fascia covering the first dorsal interosseous muscle on its ulnar edge. (c) Rotation of the flap just proximal to the interphalangeal joint, followed by insertion and suturing in place.

**Figure 2:**
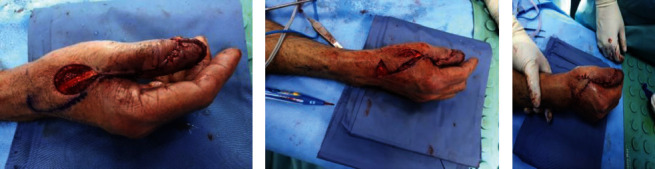
Intraoperative image showing the second and final stage of the Brunelli flap. An intraoperative image showing (a) donor defect with the marking of the Hatchet flap. (b) Rotation of the (Hatchet) flap. (c) Closing the donor site through the flap in the first webspace.

The Brunelli flap was marked, as well as the palmar connection between the dorsoulnar artery and the palmar ulnar digital artery (branch of Princeps Pollicis artery) at the level of the thumb's interphalangeal joint. The flap's axis was between the ulnar midlateral line and the dorsal middle line of the thumb's dorsum aspect, where the dorsoulnar artery was identified roughly one centimeter ulnar to the thumb's dorsal middle line (Figure 1(a)). Under awake local anesthesia without the use of tourniquet, radical excision of the nail bed was done, including both the eponychium and the periosteum, followed by the use of a high-speed burr to shave the dorsal bone and ablate the germinal matrix to prevent future nail growth. The flap was outlined just proximal to the metacarpophalangeal joint of the thumb; the dissection started from proximal to distal; on the radial edge, the skin paddle was incised till the paratenon of the extensor pollicis longus was encountered, in which it was kept intact along with the tendon; and on the ulnar edge, it was incised deep to the fascia covering the first dorsal interosseous muscle (Figure 1(b)). To avoid interfering with the underlying vasculature, the flap was elevated en bloc while keeping the pedicle approximately 1 cm wide. The flap was rotated, inserted, and sutured into place while keeping the pivot point at least 5 mm proximal to the interphalangeal joint crease (Figure 1(c)). Finally, to close the donor site, a rotational advancement (Hatchet) flap was designed in the first webspace to avoid the need for skin grafts (Figures 2(a)–2(c)).

**Figure 3:**
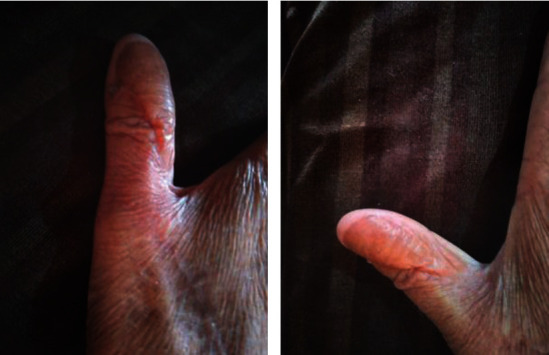
Follow-up images 2.5 years from operation. Follow-up image after 2.5 years showing the dorsal thumb webspace with complete healing of the defect and an acceptable flap appearance.

The patient was discharged home on postoperative day two, and during his stay, the flap was well vascularized. On subsequent clinic follow-up appointments, the flap did not show any evidence of ischemia or congestion. The thumb movements were full, and the first webspace was preserved unaltered. We observed no evidence of local or distant recurrences with a good functional outcome after 2.5 years of follow-up (Figure 3).

### 2.2. Patient 2

The second patient is a 54-year-old, right-handed, and retired male, nonsmoker, medically free, who underwent hemorrhoidectomy 20 years ago, with a positive family history of malignancy, esophageal cancer, in his sister. The patient was known to have a subungual right ring finger black discoloration since childhood without any change; a few months before presentation, he noticed nail pain associated with deformity; he sought medical advice and underwent nail avulsion with nail bed biopsy; the biopsy was reported as acral lentiginous melanoma.

**Figure 4:**
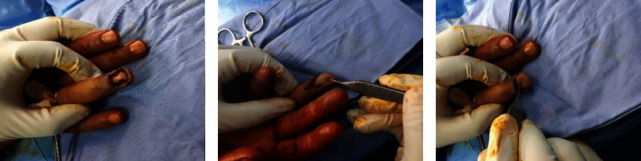
Intraoperative image showing V-Y plasty coverage of the defect. Intraoperative image showing (a) excision of the nail bed including both the periosteum and the eponychium up to the base of the nail fold. (b, c) A surprising degree of V-Y flap advancement resulting in coverage of the defect.

The patient was referred to KHCC for further management. Upon pathology review, the diagnosis was confirmed with positive resection margins, though technical difficulty was encountered to assess the exact depth of the tumor. Upon examination, nail avulsion was apparent in the right ring finger with hypertrophic changes at the nail bed, black/grey discoloration, and three black spots on the tip of the affected digit; clinically, there were no palpable lymph nodes, and upon ultrasound, there were also no suspicious axillary lymph nodes; he had Hb of 14.5 g/dl, WBC count of 6.5·10^3^/*μ*l, platelets of 124·10^3^/*μ*l, creatinine of 1.05 mg/dl, and albumin of 4.71 g/dl. Brain MRI was done and showed no metastasis; also, a local hand MRI revealed heterogeneous enhancement of the fourth digit dorsally, though no solid masses nor destruction of osseous was seen. PET/CT showed no evidence of suspicious lymph nodes or any other metastatic disease. After counseling the patient and his family, based on the MDC decision, he was scheduled for radical nail bed resection including both the eponychium and the periosteum, bone shaving of the dorsal bone of the distal phalanx, and flap coverage using V-Y plasty combined with proximal nail fold advancement flap. Even though we offered an axillary sentinel lymph node biopsy, the patient declined the latter because he was concerned about the possibility of lymphedema development. Under awake local anesthesia, after planning our flap, radical excision was done of the nail bed including both the periosteum and the eponychium as it was detached along its entire width up to the base of the nail fold (Figure 4(a)), proximal nail fold was elevated and advanced as a flap to be combined with V-Y plasty to aid in coverage, and a high-speed burr was utilized to shave the dorsal bone as well as to ablate the germinal matrix to prevent any future growth of his nail with caution not to injure the insertion of the extensor tendon. The flap was designed with a V incision that ended at the center of the distal interphalangeal (DIP) joint volar crease. The V's limbs extended down to the bone from the ulnar and radial corners of the resected nail bed. At the apex of the V, we incised through subcutaneous tissue until we reached the flexor digitorum profundus tendon sheath, where it was protected. The fibrous septa were sharply divided while sparing the neurovascular structures. Interestingly, there was a lot of flap advancement, which was sutured to the advanced proximal skin fold flap (Figures 4(b) and 4(c)).

**Figure 5:**
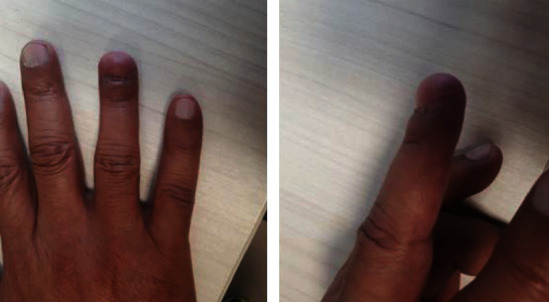
Follow-up images 2 years from operation. Follow-up image 2 years postoperative showing a dorsal and lateral view of the ring finger with an acceptable flap appearance.

Pathology came back as a minimally invasive malignant melanoma. The patient was discharged home on postoperative day one with optimal recovery. He is kept on regular follow-up for 2 years, and his latest imaging showed no disease recurrence or metastasis with intact functionality (Figure 5).

## 3. Discussion

SUM diagnosis is quite often delayed due to the subtle onset of presentation, with SCC and malignant melanoma comprising the major subtypes of such diagnosis. Given the fact that nail bed SCC is a rare entity, it is frequently misdiagnosed as a benign condition till the time of diagnosis to be associated with a significant local invasion, with the distal phalanx being involved in 20% to 50% of cases, necessitating amputation once the bone invasion is detected [3]. Also, it was found to have a 1.6% rate of distant metastasis at the time of presentation, of which 40% were fatal [4]. Subungual melanoma forms only between 0.7 and 3.5% of all melanomas, occurring in older individuals with late-onset of presentation [5, 6]. Even though digit conserving surgery has been trending for “in situ” SUM, showing no increase in the rate of local recurrence or distant metastasis [7, 8], still, the cornerstone treatment for such cases is widely accepted to be radical amputation of the phalanx proximal to the site of the SUM [9]. If nail bed invasion is detected, the same rule applies [10]. A more tissue-preserving amputation has been attempted by many surgeons, at the level of the DIP in fingers and the interphalangeal (IP) for the thumb resulting in an equal survival rate when compared to the radical resection at the level of the proximal interphalangeal (PIP) joint or the metacarpophalangeal (MCP) joint for the thumb for the “in situ” SUM [11, 12]. Even a more digit-sparing approach by using wide local excision for the “in situ” SUM has been attempted instead of the distal amputation showing similar results. It should be noted that the jury's still out on the safety for this “conservative” approach for the “in situ” SUM [10].

Because preservation of digit length in SUM is associated with higher patient satisfaction, reduced disability, and improved cosmesis, digit conserving excision is regarded as a more desirable alternative to radical amputation [8, 13].

The most common reconstructive method over the exposed bone following wide local excision for in situ SUM is a full-thickness skin graft [14–16]. That being said, it was noted that skin grafts over such areas are considered unstable closure leading to failure and break down secondary to pressure, nail spicules, inclusion cysts, persistent hypersensitivity, and persistent moderate pain [17]. Despite a wide variety of flaps being commonly used to cover fingertip traumatic defects, their use in SUM is limited [12, 18–20]. Lee et al. utilized a free superficial circumflex iliac artery perforator (SCIP) flap in reconstructing subungual melanoma for 41 patients with satisfactory results [21].

The pseudoisland flap, as it was described by Brunelli et al. in 1999, demonstrated the unique developed dorsal arterial supply to the thumb. The retrograde distal blood supply of the Brunelli flap “harvested from the dorsoulnar skin covering the MCP joint of the thumb,” allows for easy coverage of the thumb distal end [20]. Brunelli flap with its constant dorsoulnar vessels provides a reliable option for closure with minimal scarring covering “like with like.” Our goal was to maintain as much thumb length as possible to preserve hand function, knowing that loss of function of the hand is estimated to be 10% following amputation of the thumb at the interphalangeal (IP) joint and 40% following amputation at the MCP joint [22].

The V-Y plasty has been used commonly in covering defects of fingertip injuries; despite its being considered a simple procedure, it poses a significant challenge [23–26]. Regardless of its satisfactory outcome and good sensory recovery, closure under tension remains a problem resulting in tissue necrosis and flap failure [27]. In our case, flap tension was avoided by suturing it to the advanced proximal skin fold flap, which prevented the flap from moving too far.

Both of our presented patients had a noncomplicated postoperative course and have been in remission for 2.5 years for the first case and 2 years for the second. Both have demonstrated full range of motion for the affected digit and hand functionality without any limitation, redeeming both methods as an acceptable surgical option for nonbone-invasive subungual malignancy.

## 4. Conclusion

Although invasive SUM is uncommon, it is critical to consider malignancy in the presence of persistent symptoms that do not respond to treatment to avoid delay in diagnosis and prevent disease progression. Amputations are associated with long-term socioeconomic and psychological disabilities; due to that, curative surgical treatment should focus on avoiding amputations if possible, particularly in the thumb. The safety of this procedure could be confirmed by larger series and longer follow-up periods, to be able to determine whether a conservative “digit-sparing” method is equivalent to amputation.

## Abbreviations

SUM: Subungual malignancy
BMI: Body mass index
SCC: Squamous cell carcinoma
KHCC: King Hussein Cancer Center
Hb: Hemoglobin
WBC: White blood cell
MRI: Magnetic resonance imaging
PET/CT: Positron emission tomography/computed tomography
MDC: Multidisciplinary clinic
DIP: Distal interphalangeal
MCP: Metacarpophalangeal
SCIP flap: Superficial circumflex iliac artery perforator flap
IP: Interphalangeal.
